# Understanding older peoples’ chronic disease self-management practices and challenges in the context of grandchildren caregiving: A qualitative study in rural KwaZulu-Natal, South Africa

**DOI:** 10.1371/journal.pgph.0000895

**Published:** 2022-09-30

**Authors:** Dumile Gumede, Anna Meyer-Weitz, Anita Edwards, Janet Seeley

**Affiliations:** 1 Centre for General Education, Durban University of Technology, Durban, South Africa; 2 Africa Health Research Institute, KwaZulu-Natal, South Africa; 3 School of Applied Human Sciences, University of KwaZulu-Natal, Durban, South Africa; 4 Department of Global Health and Development, London School of Hygiene and Tropical Medicine, London, United Kingdom; Indian Council of Medical Research, INDIA

## Abstract

While chronic diseases are amongst the major health burdens of older South Africans, the responsibilities of caring for grandchildren, by mostly grandmothers, may further affect older people’s health and well-being. There is a paucity of information about chronic disease self-management for older people in the context of grandchildren caregiving in sub-Saharan Africa. Guided by the Self-Management Framework, the purpose of this qualitative methods study was to explore the chronic disease self-management practices and challenges of grandparent caregivers in rural KwaZulu-Natal, South Africa. Eighteen repeat in-depth interviews were carried out with six grandparent caregivers aged 56 to 80 years over 12 months. Thematic analysis was conducted based on the Self-Management Framework. Pathways into self-management of chronic illnesses were identified: living with a chronic illness, focusing on illness needs, and activating resources. Self-perceptions of caregiving dictated that grandmothers, as women, have the responsibility of caring for grandchildren when they themselves needed care, lived in poverty, and with chronic illnesses that require self-management. However, despite the hardship, the gendered role of caring for grandchildren brought meaning to the grandmothers’ lives and supported self-management due to the reciprocal relationship with grandchildren, although chronic illness self-management was complicated where relationships between grandmothers and grandchildren were estranged. The study findings demonstrate that grandchildren caregiving and self-management of chronic conditions are inextricably linked. Optimal self-management of chronic diseases must be seen within a larger context that simultaneously addresses chronic diseases, while paying attention to the intersection of socio-cultural factors with self-management.

## Introduction

While chronic diseases affect all age groups, ageing increases the risk of chronic conditions [1] such as hypertension, diabetes, and ischaemic heart disease [2] and is associated with physical functional decline [3]. The number of older people aged 50 and above living with chronic conditions continues to rise rapidly in South Africa [4, 5]. In a recent population cohort study of older, rural, black South Africans, approximately 71% had two or more chronic illnesses [5], placing a heavy burden on the South African healthcare system [3, 6].

Chronic diseases are also combined with a high burden of HIV in South Africa [7, 8]. South Africa has 7.5 million people living with HIV, with nearly 5.2 million on antiretroviral therapy (ART) [9] and more individuals are ageing with HIV in South Africa [4]. Living with HIV in the context of ART is another chronic illness burden faced by older people as effective treatment enables people to live with HIV into older age [10].

While chronic conditions pose a health burden for older South Africans [5], at the same time many act as primary caregivers for their grandchildren. The grandparent caregivers assume responsibilities associated with caregiving for their grandchildren which include providing shelter, food, and clothing for themselves and for their grandchildren [11]. As parental figures, grandparents serve as role models, provide their grandchildren with love and support, discipline their grandchildren, and impacting values to their grandchildren [12]. Different situations exist which explains why grandparents are providing caregiving to their grandchildren. Historically, grandchildren caregiving has always been common for grandparents as they have been identified as a key family support system in ensuring a critical safety net for children [13]. However, the HIV epidemic further aggravated the responsibility of grandparents for their grandchildren due to the increased parental morbidity and mortality [14]. While HIV care and ART services have significantly reduced AIDS-related deaths, grandparents still assume caregiving responsibilities for the grandchildren [15]. In South Africa, labor migration and unemployment have also contributed to the caregiving of grandchildren by grandparents [11]. The responsibilities of caring for grandchildren combined with living with chronic conditions may further impact the health and well-being of grandparents [16] and this may be physically, economically, psychologically, and socially a burden for the grandparents. In Uganda, older grandparent caregivers reported chronic pains and stress as major challenges in their role as carers, thereby limiting their ability to effectively execute caring duties [17]. This caregiving has been linked to worse mental health issues and social isolation for the grandparent caregivers [18].

Grandchildren caregiving is often undertaken with minimal financial resources [15]. In response to supporting poor families, the South African government offers social security grants including old-age pensions, child support grants, and foster child grants to eligible grandparents, which they can claim for themselves and their grandchildren [12]. It has been reported that these government grants are important sources of income, especially for grandmothers raising grandchildren in South Africa [11, 19].

When discussing the caregiving provided by grandparents, it is worth noting that gender differences shape caregiving within families [20]. The literature pertaining to grandchildren caregiving shows that the caregiving role is performed predominantly by women [15, 21]. Given that it is normative in the South African context for grandmothers to provide care for grandchildren [11], this further contributes to the notion of gendered caregiving of grandchildren [12, 20]. While there is little available research globally on the number of grandparents raising children, a study in Uganda among grandparent caregivers reported that the majority of caregivers of children aged 13–17 years were grandmothers aged 50 years and older [17]. In 2019, it was estimated that nearly 4 million children in South Africa were living with a grandparent [22]. Older grandparent caregivers with chronic conditions are likely to experience less time for self-management because, as previous research has established, chronic disease affects the ability of older people to function [3, 10]. Self-management is defined as the individual’s ability, in conjunction with family, community, and healthcare professionals, to manage symptoms, treatments, lifestyle changes, and psychosocial, cultural, and spiritual consequences of chronic conditions [23].

Understanding how grandparent caregivers of adolescents navigate the self-management of chronic conditions while at the same time taking on the added responsibility of caring for their grandchildren is a neglected research area. The personal health and well-being challenges of grandparent caregivers are likely to take a toll not only at a personal level, but also impact the caring process for adolescents in different ways. In this study, we explored the chronic disease self-management practices and challenges of grandparent caregivers and their views on how this was shaped by caring for adolescent grandchildren. The Self-Management Framework was used to shape the analysis because it can be applied across a wide range of individual characteristics and chronic conditions [24]. Self-management activities cluster into three main processes: focusing on illness needs, activating resources, and living with a chronic illness, each of which includes tasks and skills that facilitate self-management [25]. Understanding factors that influence self-management may improve the assessment of self-management among grandparent caregivers with chronic illness and inform interventions to support older caregivers.

## Methods

### Study context

The study was conducted in uMkhanyakude district (KwaZulu-Natal province, South Africa), one of the poorest and rural districts in the country [26]. People in the district live in predominantly multi-generation families consisting of grandparents, adult children, and grandchildren [27]. The area is among those with the highest HIV prevalence and incidence rates in South Africa [28]. Between 2009 and 2015, the prevalence of hypertension, HIV, and diabetes increased in the district [29]. While there is a high burden of chronic illness, there are only five district hospitals, 251 primary care clinics, and 17 mobile clinics servicing a population of 625,846 [30]. Approximately 10% of households in the district can reach a primary healthcare facility within 15 minutes by vehicle [29]. A previous study, undertaken two decades ago, reported that livelihood strategies of most households depended on small-scale agriculture, government grants, and remittances from migrant workers [31]; the situation has remained the same to the present time.

This study was part of a larger study at Africa Health Research Institute (AHRI) that commenced in September 2017 investigating the caring of adolescents by grandparent caregivers in the context of DREAMS (Determined, Resilient, Empowered, AIDS-free, Mentored and Safe) partnership. DREAMS is a multi-component HIV prevention intervention designed to reduce HIV incidence among adolescent girls and young women (AGYW) [32, 33]. The DREAMS programme was implemented between April 2016 and September 2018 and delivered by different DREAMS implementing partners in the district.

### Study design, study population and sample

A qualitative research design located within an interpretive paradigm was adopted for this study because of the focus on exploring the experiences of grandparent caregivers and the meanings they attribute to self-management of chronic diseases whilst caring for grandchildren. This design has proven useful in previous research on chronic illness [34, 35] and we sought to generate depth accounts. Participants were selected using a purposive sampling method with the following criteria: grandparents aged 50 years and above who were primary caregivers of at least one adolescent child aged 13 to 19 years who participated in DREAMS programme.

### Data collection

The first author, a local social science researcher trained in qualitative methods, conducted eighteen repeat in-depth interviews with six grandparents from uMkhanyakude district. The grandparents were recruited through a community-based organisation that delivered DREAMS interventions and were primary grandparent caregivers of adolescents that had received DREAMS interventions. The repeat in-depth interviews were conducted on three occasions from September 2017 to October 2018, using a semi-structured interview guide. The first interview focused on two central, open-ended questions that were posed to each participant: “How do you care for yourself?” and “How is it for you to raise your grandchildren while caring for yourself?” As the participants reflected on their experiences, the interviewer began to identify the challenges that they were experiencing with chronic illnesses while raising their grandchildren and the manner in which these influenced their lives. The repeat interviews were conducted every four months to maintain contact with research participants over time. As opposed to single interviews, repeat interviews are useful for documenting participants’ lived experiences over time, allowing researchers to ask follow-up questions and can be tailored for each individual [36]. After the first interview, data were preliminary analysed to identify emerging themes and issues to follow-up with each individual in the subsequent interviews. Through these repeat in-depth interviews, we were able to capture the daily lives, the caregiving experiences of participants, the experience of ageing, and health challenges. Also, prolonged engagement strengthened rapport that was established with participants and increased trustworthiness in data collection. Interviews were carried out in the local language, isiZulu. The grandmothers opted that interviews be conducted at their homes. This ensured privacy and confidentiality. Each interview took between 30 and60 minutes.

### Data management and analysis

Interviews were audio-recorded, transcribed, and translated into English. Atlas.ti 8 software was used to manage, and code translated data. The Self-Management Framework [24] guided the thematic analysis of data, using deductive and inductive processes. The analysis followed a five-step process, as described by Babchuk [37], that involved assembling materials for analysis; structured reading and re-reading of transcripts; coding the data along the identified themes of the self-management framework; generating categories and assigning codes to them; generating themes from categories; and using verbatim quotes from the in-depth interviews to illustrate certain themes. Coded data, themes, and categories were repeatedly reviewed by DG and agreed with AMW and JS to increase the rigour of the analysis and to ensure a deeper understanding of the data.

### Ethical considerations

Ethics approval for the study was provided by the University of KwaZulu-Natal Humanities and Social Sciences Research Ethics Committee (HSS/1109/017D). Voluntary written informed consent was obtained from participants after information about the purpose of the study had been provided. Confidentiality and anonymity were maintained through the use of participant codes and pseudonyms when the participants referred to their grandchildren during the interviews. All identifying information were removed from quotes.

## Results

All six participants were grandmothers aged between 56 and 80 years old and primary caregivers of two to fifteen grandchildren, at least, one of whom was an adolescent child aged 13 to 19 years and a recipient of DREAMS. While we did not collect data about all biological parents of the grandchildren that were raised by grandmothers, the biological fathers of four adolescent grandchildren who were recipients of DREAMS were deceased and the biological mothers of all six grandchildren were still alive. None of the adolescent grandchildren receiving DREAMS had both biological parents deceased. Chronic conditions varied widely between participants with all of the participants reporting living with one or more chronic conditions. Most grandmothers relied on the government’s social security grants including old age pension (approximately $100 per month) and child support grant (approximately $24 per month) and survived by growing and selling produce, even though it was on a small scale. The demographic characteristics of the participants, including health conditions, are presented in Table 1.

**Table 1:** Participants’ demographic characteristics and chronic conditions.

Participant Code	Age	Marital status	Education	Number of children in care	Source of income	Chronic conditions
P1	76	Widow	None	3	Old age pension, cash, or in-kind remittances	Arthritis, chronic pain
P2	64	Widow	Secondary	15	Old age pension, child support grant	Arthritis, hypertension, chronic pain
P3	80	Widow	None	6	Old age pension, informal micro-enterprising	Arthritis, hypertension, stomach ulcers, chronic pain
P4	58	Unmarried, living with partner	None	11	Child support grant, informal micro-enterprising	Chronic pain
P5	56	Unmarried	None	2	Farm work, informal micro-enterprising	HIV, epilepsy, chronic pain
P6	64	Widow	Primary	9	Old age pension, child support grant	HIV, hypertension, vision impairment, chronic pain

### Chronic disease self-management practices and challenges

The findings are structured according to the processes drawn from the Self-Management Framework [24] (Table 2), namely living with a chronic illness, focusing on illness needs, and activating resources. The quotations included throughout the results section are all from different participants’ responses.

**Table 2:** Themes and sub-themes for chronic illness self-management by grandmothers.

Themes	Sub-themes	
Living with a chronic illness	Processing emotions	• Feeling shocked, confused, sad, and angry • Blaming self for acquiring HIV
	Adjusting to illness and to a new self	• Accepting and embracing the illness as part of ageing • Comparing self to others as a way of fostering self-motivation
	Integrating illness into daily life	• Reorganising everyday life in order to adapt to chronic illness
	Meaning making	• Illness as a pathway to end of life • Caring for grandchildren as sense of purpose
Focusing on illness needs	Following instructions from the healthcare workers	• Treatment adherence • Eating healthy diet • Regular clinic attendance
	Completing health tasks	• Regular medical appointments • Collecting treatment from the facilities • Adhering to treatment
	Performing health promotion activities	• Using home remedies, traditional medicine, and alternative therapy
Activating health resources	Healthcare resources	• Regular interactions with community healthcare workers • Seeking care from multiple healthcare facilities
	Spiritual resources	• Regular church attendance • Praying
	Family support	• Home remittances from adult children • Assistance with performing household tasks by grandchildren • Support with taking medication by grandchildren
	Community resources	• Participation in community saving clubs

#### Living with a chronic illness

The grandmothers explained how living with a chronic illness impacted on their lives and required them to come to terms with their conditions. They had to manage their emotions following their chronic illness diagnoses and then make any required adjustments in daily living.

Firstly, the older carers narrated stories about the diagnosis of their chronic illness(es) and how they **processed the emotions** of living with a chronic illness as the narratives of two grandmothers illustrate:

*Sometimes I say ‘oh hee!’ [yelping] and then tell myself to accept the situation … the eye doctor said ‘your eyes are dirty inside the eyeball. There are dark marks, I said ‘please wipe those dirty marks’ as I’ve heard that doctors can wipe eyes. The doctor said, ‘it’s not going to make any difference even if we can try to wipe your eyes because it’s inside*. *You must just go home and expect to die like this. (P6, Female, 64yrs)*

*If I think of eating boiled and unsalted food*, *I simply lose appetite. Unsalted food is tasteless. But what can I do because I have BP [high blood pressure] and the nurses are preaching that we should boil food and not use salt? (P3, Female, 80yrs)*

Living with HIV and lacking financial resources to self-manage, generated feelings of self-blame for acquiring HIV infection, and helplessness among the grandmothers:

*I didn’t know that involving myself with men was a problem. I thought I was just having a good time, whereas I took a burden upon my shoulders* [meaning HIV infection]. *(P5, Female, 56yrs)*

*We don’t use an electric stove*, *but firewood for cooking because electricity is expensive, and I’m financially struggling. (P2, Female, 64yrs)*

However, one grandmother compared herself to other people living with HIV as a way of fostering self-motivation, as she stated:

*There was a woman at* [name of place] *who stopped taking ART and eventually died. I don’t want to die like her, that’s why I’m taking my pills every day. The reason I’m still alive is that I’m taking my pills. (P5, Female, 56yrs)*

The grandmothers also shared their initial experiences of the **adjustments they had to make because of the illness and to come to terms with the ‘new self.’** They identified two strategies that they used to adjust to the illness and their new self: accepting and embracing the illness as part of ageing and comparing themselves to others as a way of fostering self-motivation.

One grandmother talked about how she had expected that her life would deteriorate because of ageing and the illness:

*My life is now weakening*, *and I tell myself that it’s because I’m ageing. I can feel my body is unlike the one I had during my early days. I feel it when doing chores; I am not strong as I was. … Even walking, I cannot walk like a fit person. (P6, Female, 64yrs)*

Seasonal weather conditions also affected the physical well-being of the grandmothers as they were unable to function optimally. In some instances, living with a chronic illness was very limiting:

*As it is now a cold season*, *I’m unable to wake up early morning. My joints are stiff and painful. I’m horribly inactive in the morning and would take time to recover. (P2, Female, 64yrs)*

When describing life with chronic illness, the same participant, P2, recounted the strategies she employed to **integrate illness into her daily life** by reorganising her life to adapt to living with arthritis:

*In winter*, *I don’t touch cold water because that makes my bones sore. I also don’t do handwashing of clothes when it’s very cold. I only do the washing when the sun is warmer. Also, I wear warm clothes like warm jackets, so that my skin would not feel cold. (P2, Female, 64yrs)*

Our data suggest that grandmothers’ focused on the challenges of chronic illnesses in their capacity to function and on the disruptions that the chronic illnesses brought to their lives.

Among the accounts of living with a chronic illness, **meaning making** about the chronic illnesses featured during the interviews. P6 described illness as a pathway to end of life:

*I often tell Mpume [*granddaughter*] that I might die soon because I am old and sick. Living with all these diseases* [HIV, vision impairment, hypertension, and chronic pains] *means my life will be shorter than others. (P6, Female, 64yrs)*

Some mentioned that they found a sense of purpose in life through caring for their grandchildren. Grandmothers perceived themselves as caregivers. Caring for their grandchildren was more important than receiving better care for themselves:

*I may be sick but I’m still looking after my grandchildren*. *It gives me a reason to wake up in the morning. Who will look after my grandchildren if I go to an old age home? Being sick doesn’t mean I cannot talk. I can still talk and could still guide them about life. (P1, Female, 76yrs)*

*I can’t* [go to an old-age home]. *Who am I going to leave them with? It’s better to suffer with them. (P3, Female, 80yrs)*

Meeting needs such as food for the grandchildren was also performed by the grandmothers. Instead of focusing on managing their chronic illnesses, grandmothers opted to prioritise the needs of their grandchildren:

*They are still young*; *they deserve to eat as much as they like. I’m about to die. Even if I sleep with an empty stomach, I am fine knowing that they have eaten. (P3, Female, 80yrs)*

*Now that Mpume* [granddaughter] *is pregnant, I will be looking after her baby so that she would go back to school. When am I going to find time to rest? I need to rest too but who will look after Mpume’s baby because her* [biological] *mother disappeared. (P6, Female, 64yrs)*

However, while grandmothers valued meeting the needs of their grandchildren instead of focussing on themselves and their use of resources to self-manage their chronic conditions, regret was also felt because of the additional responsibilities and consequences of caring for their grandchildren.

*I do care for myself*; *however, I’m unable to do this well because I have so many grandchildren. I wish I could eat nice things; but I can’t. I wish I could go to doctors to get booster injections so that I could live a healthy life. I can’t get to eat nice things like other older people. I can’t afford to stock food items like apples, bananas, yogurts, and meat all the time. No, I can’t because I have so many grandchildren. We use a big pot for cooking food which gets finished in no time. I’m unable to care for myself properly because of the large number of grandchildren. I’m not eating enough food. (P2, Female, 64yrs)*

Our data suggest that grandmothers struggled to find access to their desired proper healthcare, availability of food, and their ability to access nutritious food as they were living with chronic conditions.

#### Focusing on illness needs

When grandmothers focused on managing their chronic illness needs, they indicated a range of activities that they perform.

The grandmothers mentioned that they were **following instructions from the healthcare workers** about the management of chronic conditions. These instructions were related to treatment adherence, a healthy diet, and regular clinic attendance for routine check-ups:

*At first*, *I was diagnosed with TB and was taking pills for six months. I never missed taking my pills until the end. Now, I’m taking HIV pills and am making sure that I always have my pills. (P5, Female, 56yrs)*

Fear of being reprimanded by the nurses for not adhering to these instructions appeared to motivate the older people in ensuring that they followed dietary requirements:

*We are told to eat healthy foods at the clinic*. *The nurses make us choose healthy food to eat. They say, ‘don’t use cooking oil. Eat boiled food.’ They say, ‘salt makes us sick.’ We now eat food that is not tasty. Nonetheless, I do follow the instructions from the clinic because nurses can see if we don’t follow the instructions. (P3, Female, 80yrs)*

However, the ability of some grandmothers to take ownership of their health needs by following appropriate meal plans was limited by their adolescent grandchildren refusing to cook healthier meals or to provide needed support for healthy eating:

*It’s sometimes difficult to maintain a healthy diet when you don’t cook for yourself. Thandi* [granddaughter] *doesn’t want to boil food. She likes frying food with excessive cooking oil. Fried food is not good for me. The nurses educate us to eat boiled vegetables instead of frying them … I would eat fried food because I have no choice. Sometimes, I opt to cook my own food with a small pot so that I don’t keep on eating unhealthy food … [however] I normally avoid cooking because of epilepsy. I don’t want to have seizures while I’m next to the stove. (P5, Female, 56yrs)*

*I have a vegetable garden in the backyard, where I plant pumpkins, mealies, cabbages, nuts, and beans. Fresh vegetables are healthy unlike those that we buy from the shops. My problem is Thabani* [grandson]. *He refuses to go and fetch water for me to water the garden. If there is no rain, my vegetables get destroyed for not getting water. Sometimes, I hire* [name] *to fetch water for me because I cannot push a wheelbarrow nor carry a 25L bucket on top of my head. (P1, Female, 76yrs)*

While refusal to cook healthy food was mentioned as one of the main challenges that grandmothers faced with focusing on illness needs, another grandmother related how living with poor vision as a chronic condition limited her independence and her ability to eat healthily:

*I’m taking my treatment after eating food as advised by the nurses and visit the clinic for my appointments. The problem is that I can’t see properly. So, I no longer cook otherwise I would simply burn the food. Mpume [*granddaughter*] and others are the ones cooking. The problem is that they don’t like cooking traditional food like ‘amadumbe’ and samp with crushed nuts. I like traditional food because it’s healthy and the nurses encourage us to eat healthily. (P6, Female, 64yrs)*

Rather than supporting their grandmothers’ efforts to eat well, the adolescent grandchildren were quite dismissive of the health concerns of their older carers. Dependence on adolescent grandchildren to prepare meals for the grandmothers presented a challenge to self-manage chronic conditions.

Grandmothers mentioned **completing health tasks** in their effort to self-manage chronic conditions. Attending regular medical appointments, collecting treatment from the facilities, and adhering to treatment were key in the management of their illnesses.

Some grandmothers indicated that they performed these health tasks independently while others relied on the support of their adolescent grandchildren. This was raised by two grandmothers living with HIV and on ART as they explained that their adolescent grandchildren supported them in taking medication and escorted them to the clinic:

*I’m on treatment for high blood pressure and HIV*. *I was first on treatment for BP and later started ART in 2011*. *I take my pills at seven in the evening*. *However*, *before I take my pills*, *Mpume [granddaughter] needs to first give me food*. *After I had eaten*, *then she gives me my pills*. *… I recently went to the clinic [*with her granddaughter*] for blood tests and they told me my blood results are excellent*! *(P6*, *Female*, *64yrs*)

*I’ve been taking ART for the past seven years*. *It’s Thandi [*granddaughter*] who reminds me every day that it’s time to take my pills*. *(P5*, *Female*, *56yrs*)

Lastly, grandmothers mentioned that they **performed health promotion activities** to minimise the impact of chronic conditions and engaged in self-initiated treatment like using home remedies, traditional medicine, and alternative therapy for their conditions. Alternative therapies and home remedies were mentioned to self-manage the treatment of chronic illnesses and minor ailments including fever, heartburn, body pains, and skin rashes. The use of alternative therapies was motivated by factors such as the lack of access to medication at healthcare facilities and standard healthcare treatments which the older carers perceived as ineffective:

*I become sick once something upsets me*. *I ended up getting herbal tea from a certain woman in the community because I was just not feeling alright at all*.… *It’s herbal medicinal tea for drinking*. *Remember*, *I take pills for BP and arthritis*, *but sometimes I don’t get arthritis medication and painkillers at the clinic*.… *The nurses said they don’t have pills for arthritis and painkillers … BP pills are for BP; they are not painkillers*. *Yesterday*, *I was literally bedridden but I feel better today*. *The herbal tea helped me*! *They say it runs through the veins and gives you energy*! *I cooked it in the morning and I already feel better*. *It also relieves pain*. *(P3*, *Female*, *80yrs*)

*The nurses at the clinic used to give me pills for epilepsy but those pills were not effective*. *I felt like they were making me worse*. *… After taking the pills*, *I would just get seizures*. *Then I decided to take the herbal tea for a while until the seizures stopped*. *(P5*, *Female*, *56yrs*)

Salt was used as a common commodity for bathing, steaming, and soaking, for the grandmothers to cope with chronic pain, and also for fever:

*My feet*, *ankles, and knees are often painful and swollen because I have arthritis. To care for myself, mostly I walk barefoot and soak my feet in warm salt water. Salt has anti-inflammatory properties that help reduce swelling. I also have some oil that I use to rub my feet. (P1, Female, 76yrs)*

*Salt is my number one defence! I use it to steam and soak my body whenever it is painful or if I have symptoms of fever. Milk is another thing that I occasionally use for heartburn. It’s just that I’m able to get cow milk if my son’s cow has given birth … I warm and drink cows’ milk for heartburn. (P3, Female, 80yrs)*.

Grandmothers often reported anxiety and emotional distress related to living with chronic conditions, the strained relationships with their adolescent grandchildren, and their poverty. Burning incense to cope with emotional ‘pains’ was mentioned by some grandmothers:

*My heart is sometimes painful*, *especially when I don’t have money and think about living with HIV. I would simply take ‘impepho’ [incense] and burn it inside my bedroom. Smoking the flames of ‘impepho’ calms me. (P5, Female, 56yrs)*

*I burn ‘impepho’ to manage emotional pains … Sane [*granddaughter*] is making me angry with the way she’s behaving. ‘Impepho’ helps me a lot otherwise my heart is just going to stop beating* [dying]. *(P4, Female, 58yrs)*

Improving musculoskeletal function and mobility through performing physical activities was seen as a benefit by all the grandmothers. They referred to physical activity as ‘*ukunyakazisa igazi*’ meaning ‘an act of moving one’s body for blood circulation in the body system.’ Walking, dancing in church, doing household chores such as gardening, washing clothes and dishes by hand, and sweeping the yard and house were the main daily physical activities that the grandmothers performed to remain physically fit.

Interestingly, P5 mentioned how she had changed a negative experience into something positive by thinking differently about it i.e., reframing the “problem”:

*I usually do garden work every morning*. *It’s just that the chickens destroy my garden because it’s not fenced, and I don’t have a chicken house to secure the chickens. Nonetheless, chasing the chickens from destroying my garden keeps me fit. (P5, Female, 56yrs)*

Directing negative emotions of living with chronic illnesses onto other activities in order to get rid of the negative feelings was a defence mechanism for the grandmothers.

#### Activating resources

In exploring the resources that the grandmothers activated, they mentioned healthcare resources, spiritual resources, family resources, and community resources to manage various aspects of their chronic illnesses.

With regards to **healthcare resources** that were important for the grandmothers, community healthcare workers (CHWs), and healthcare facilities were mentioned as key resources. The grandmothers stated that they regularly interacted with CHWs who provided home-based care and health education:

*We have community health workers visiting us at our homes*. *They tell us not to sit down for too long as we are elderly people who are also sick. We need to make the blood flow through the body by being active as elderly people. So, although my eyes cannot see anymore, I make my blood flow by working in the garden. (P6, Female, 64yrs)*

*The community health workers sit down with us when they visit our homes*. *They would advise us that no matter how difficult life is, we must make things easier for ourselves and take things lightly … by not thinking too much … As elderly people, our hearts are weak, thus, we must avoid thinking too much about our problems, otherwise, we will be attacked by stroke. (P2, Female, 64yrs)*

Grandmothers also mentioned that they found CHWs easily accessible in case of emergency as they lived in the community unlike going to the clinic to consult the nurses. Interestingly, some grandmothers indicated that they also made use of the services of the CHWs for healthcare needs related to their adolescent grandchildren:

*I asked the CHW to test Mpume [*granddaughter*] for pregnancy. I was worried because she had missed her menstrual period for two months and wanted to know whether she was pregnant or not. The CHW, then, came to my house to do the pregnancy test. She then referred Mpume to the clinic [*for antenatal care*]. (P6, Female, 64yrs)*

*The CHW helped me with a referral letter, which she gave to Zama [*granddaughter*] to go to the clinic for contraception. (P3, Female, 80yrs)*

When grandmothers described how they navigated the healthcare system in order to ensure continuity of primary healthcare services, they talked about healthcare facilities that they opted to use. Grandmothers mentioned that they seek care from multiple healthcare facilities using both the provincial and the local government clinics interchangeably:

*For BP*, *I go to [clinic X] to collect my medication, whereas for arthritis I prefer to go to [clinic Y]. There at [clinic X], sometimes, the nurses tell you that the pills are out of stock. (P3, Female, 80)*

*I normally go to [clinic X] because it’s closer to us than [clinic Y]*. *(P5, Female, 56yrs)*

*There is this nurse at [clinic Y] who is so caring whenever she attends an older person*. *If she is not on duty, I sometimes go back home and return on another day when she’s back at work. She is such a caring nurse and gives you all the pills that you need. … Others simply tell you that the pills are out of stock or give you insufficient pills. (P4, Female, 58yrs)*

It was clear that the quality of patient care, specifically the lack of a patient-centered approach, distance to the healthcare facility, and availability of medication influenced the grandmothers’ choices of the healthcare facilities to use for their chronic illnesses.

Moreover, **spiritual resources** were a key component of health and well-being that the grandmothers mentioned that sustained them. They all mentioned that they regularly attended church services to worship and receive spiritual counseling. In addition, praying was a source of strength:

*They* [pastors] *preach to us that we mustn’t overburden our hearts with worries to prevent stroke. (P2, Female, 64yrs)*

*Whenever I am at church*, *I become alive again. I forget about all the illnesses and all the problems that Zama [granddaughter] brings to my life. (P1, Female, 80yrs)*

Churches provided valued support to manage chronic illnesses and difficult relationships with their adolescent grandchildren.

Some grandmothers mentioned that they obtained **family support** in a form of home remittances from their adult children to cover the costs of self-managing their chronic illnesses and to meet the basic needs of their grandchildren:

*My eldest daughter is helpful. Although she is unemployed, she sells meat in town… to afford to buy us food. (P5, Female*, *56yrs)*

With regards to household chores, all the grandmothers mentioned that they sought assistance with performing household tasks from their adolescent grandchildren. They assigned household chores to the grandchildren such as cooking, cleaning, fetching water and wood as they were often sick and physically unable to conduct the housework themselves.

*I don’t usually cook anymore*. *My grandchildren do the cooking. The grandchildren go out to fetch firewood from the forest. They are so helpful. Having grandchildren means that I can point out where I need help and they would help me. (P2, Female, 64yrs)*

Yet, while some grandmothers received support from their adolescent grandchildren, they were not always satisfied with that support:

*A child must be sent to do things for the adults at home. Thabani [*grandson*] must wash dishes for me. He must fetch water for me. But he is very lazy. (P1, Female, 76yrs)*

*I no longer have physical strength*. *I find myself crawling on my knees to prepare food, while Zama [granddaughter] has gone out. Yet, she would want food when she comes back at night … then I have to cook because there are younger grandchildren who need food to eat, just like myself. So, I make fire and cook. (P3, Female, 80yrs)*

The grandmothers were distressed when their adolescent grandchildren did not meet their expectations.

Lastly, mobilising **community resources** through participation in community-saving clubs including burial schemes, grocery schemes, and loan schemes was also mentioned by the grandmothers. These clubs provided quick cash loans to the older carers to meet their needs to manage chronic illnesses and to care for their grandchildren, as seen in the interviews below:

*I borrow money from the loan scheme to buy food*, *to go to a doctor or to buy clothes for the grandchildren. (P2, Female, 64yrs)*

*I’m a member of a burial scheme with the hope that if I die or my family member dies*, *the burial scheme will cover the funeral costs. We also borrow money from the burial scheme and pay it with interest. I prefer borrowing from our scheme because the interest rate is very low. It is much quicker to get money from the scheme because we need quick cash to buy food. (P3, Female, 80yrs)*

Community-saving clubs facilitated access to micro-finances for the grandmothers to support their chronic disease self-management practices.

## Discussion

This study has uncovered three processes that shape the lived experiences of chronic disease self-management by older grandparent caregivers in a rural community of KwaZulu-Natal. Self-management involves the tasks that people living with a chronic illness must do to gain control of their condition and to live successfully with the chronic disease [24]. Living with a chronic illness, focusing on illness needs, and activating resources are the processes employed by grandmothers for their chronic disease self-management practices. Self-perceptions of caregiving dictated that grandmothers are responsible to assume primary caregiving for grandchildren when they themselves needed care, lived in poverty and with chronic illnesses that require self-management. The gendered role of caregiving for grandchildren brought meaning to life and supported self-management due to the reciprocal relationship with grandchildren. However, chronic illness self-management was complicated where relationships between grandmothers and grandchildren were strained.

This study expands on previous studies by contributing to knowledge about caregiving experiences of grandparent caregivers living with HIV and the intersection of meaning making of illness and caring [4, 38, 39]. Having grandparent caregivers with chronic illnesses could have a significant impact on the adolescents and the care that the adolescents receive from their grandparent caregivers. HIV stigma by association is one of the challenges that may face adolescents who are being raised by HIV-positive grandparent caregivers. Studies in high-income settings reported that adolescents with HIV-positive parents perceived themselves as different from their peers or feared they would be discriminated against if their parents’ HIV status is disclosed [40–42]. In a South African study, adolescents with HIV-positive caregivers were reported to have increased risks of poor educational outcomes, mental health problems, stigma, and isolation from peers [43].

It is critical for older people to engage in physical activity to prevent diseases, maintain independence, and improve their quality of life [44]. Other authors have noted that physical activity programmes target younger people more than older people and it is also less accessible for older people due to smaller incomes [45]. Our findings show that older people can use minimal resources to meaningfully engage in physical activities without financial costs and within their home environments. Consistent with previous studies, working around the house as part of domestic responsibilities was viewed as a form of exercise [46]. Apart from engaging in physical activities to prevent immobility, it is possible that physical activity also improved their mental health as they were often distressed by living with chronic diseases and caring for grandchildren. Our findings show that the impact of living with chronic illness(es) and caring for adolescent grandchildren had a toll on the health and well-being of the grandparent caregivers. The grandparent caregivers indicated mental distress in relation to living with chronic illnesses, lack of finances, and strenuous relationships with their adolescent grandchildren. Consistent with previous studies, HIV-positive caregivers and caregivers of orphaned and vulnerable adolescents are vulnerable to mental health problems [47, 48].

Mental distress is likely to impact the self-management of chronic illness(es) among grandparent caregivers. Problems experienced in everyday living have been reported to negatively impact the self-care of chronic illness [49]. Consistent with our findings, in another study among older men and women living with HIV in Uganda [50], difficult relationships with their adolescent grandchildren, rather than chronic conditions, were the main stressors that often undermined the grandparent caregivers’ ability to self-manage. This finding differs from a study conducted in the United States, where older adults with chronic conditions often did not want to burden their children with the responsibilities of caring for them [51]. In this study, grandparent caregivers expected their adolescent grandchildren to care and support them. It is possible that the caregivers’ expectations determined the relationships between grandparent caregivers and their adolescent grandchildren.

Consistent with the literature, family support is critical in sustaining self-management behaviours and addressing the barriers among people living with chronic illnesses [25, 52, 53]. Adolescent grandchildren provided support to their grandparent caregivers in executing self-management tasks such as treatment adherence, regular healthcare attendance, and cooking food. In this study, the grandparent caregivers emphasised the key role that the emotional and physical support from their adolescent grandchildren played in self-managing their chronic conditions. Support from their adolescent grandchildren facilitated the grandparent caregivers in self-managing chronic conditions. This finding is supported by other studies [17, 54] that reported the grandparent caregivers counted on their grandchildren to perform household chores that were too physically demanding for the caregivers to perform. They emphasised the vital role of social support not only in helping them to take their medication but also in helping them to find a sense of purpose in caring for their grandchildren. A study in Uganda found that adolescents supported caregivers’ adherence to HIV treatment by reminding them to take ARVs and honour clinic appointments [55]. Consistent with a study in South Africa [54], grandparent caregivers also believed that focusing on their grandchildren contributed to their sense of resilience and living with chronic illnesses.

However, adolescent grandchildren can also pose barriers to self-management behaviours for the grandparent caregivers. Dietary changes are commonly used as essential strategies to improve the self-management of chronic illnesses [56]. While adolescent grandchildren played a vital role in cooking for their grandparent caregivers, the grandparent caregivers in this study reported that grandchildren usually provided the grandparent caregivers with unhealthy food. Rather than supporting their grandparent caregivers’ efforts to eat healthily, the adolescent grandchildren were dismissive of the health concerns of the older carers. The findings of this study fill an important knowledge gap about the influence of adolescent grandchildren on dietary modifications for older carers with chronic illnesses. Previous studies have focused on the caregiver influence in relation to their children’s eating behaviours [57, 58]. In this study, grandmothers had limited financial resources to manage chronic illnesses and to provide basic needs for the grandchildren. Lack of finances negatively impacted the grandparent caregivers in self-managing their chronic illnesses in this study. The specific resources that the grandparent caregivers chose to mobilise were influenced by their human agency and the nature of their relationship with their adolescent grandchildren. For instance, participating in micro-finance activities was influenced by the caregivers’ agency and the need to provide the adolescent grandchildren with food.

Our findings add depth to previous research relating to the role of CHWs in society and particularly in supporting grandparent caregivers with the self-management of chronic illnesses. Previous studies have shown that the role of CHWs includes health education, home-based care, and supporting adherence to treatment [59, 60]. This study also reveals their role in facilitating access to and utilisation of sexual and reproductive (SRH) services by adolescents in grandparent families. For instance, it was reported that the CHWs conducted pregnancy testing, referrals for antenatal care (ANC), and referrals for contraception, thus promoting healthcare service utilisation by adolescents in grandparent families. Home visits by CHWs seemed to be effective in identifying pregnant adolescents and those needing contraceptives in grandparent families. A study in South Africa showed that healthcare providers or nurses tended to impose their values upon adolescents regarding contraceptives and posed challenges for adolescents’ uptake of SRH services [30]. It is possible that home visits by CHWs and the relationships they have with the grandparent caregivers could positively influence adolescents’ uptake of SRH services. These are in keeping with findings from another study conducted in South Africa, where older adults valued the services that they received from the CHWs [61].

Our findings resonate with other work in Malawi, Uganda, and South Africa showing how chronic patients experienced difficulty obtaining medicines from public healthcare facilities, leading to non-adherence to healthcare services [34, 35, 62]. The shortages of medicines in public healthcare facilities can also be regarded as the health systems barrier to support chronic disease self-management [34]. Being dissatisfied with shortages of medicines was one of the reasons that grandparent caregivers practiced switching healthcare facilities and it compromised chronic disease self-management.

The findings provide a framework within which services and interventions can support grandparent caregivers. Caring for grandchildren while suffering from a chronic illness can be draining and the older carers may need a combination of support to self-manage these conditions and strengthen relationships with their grandchildren.

## Strengths and limitations

Our study has made a contribution to the limited literature about the intersection of chronic disease self-management and caring for grandchildren. It has highlighted the potential of the Self-Management Framework to illuminate the complex lived experiences of navigating chronic disease self-management and caring for grandchildren. The main limitation of our study is we interviewed only grandmothers and had a limited perspective on the experience of grandfathers. While this may be an imbalance in our participants, it does reflect the gender distribution of grandparent caregivers, most of whom are women, and the gendered nature of caregiving. In addition, while our study included a small sample of grandparent caregivers living with chronic conditions, a larger sample would have been desirable and would likely have given us insight into other chronic diseases that are a burden in South African older people e.g. diabetes. Nonetheless, repeat in-depth interviews with the small sample enabled participants to provide detailed and in-depth accounts. The repeat in-depth interviews ensured data saturation which gave us confidence that we achieved diverse themes that emerged from the repeat interviews.

## Conclusions

This study provides needed information for planning primary healthcare needs and chronic care health services that will increasingly have to support older people with chronic illnesses. This evidence points to a range of self-management practices used by the older grandparent caregivers which were often influenced by the nature of care relationships between the caregivers and their adolescent grandchildren. The findings show that self-management of chronic conditions and grandchildren caregiving are inextricably linked. Health promotion researchers and health providers cannot view self-management of chronic conditions by grandparent caregivers as a single issue and hope to attain optimal health and well-being for older populations. Optimal self-management of chronic diseases must be seen within a larger context that simultaneously addresses chronic diseases, while paying attention to the intersection of social and cultural factors on older caregivers’ self-management strategies. Understanding the role played by grandchildren in supporting their grandparent caregivers’ self-management practices can assist in developing an intervention in which young people participate in self-management education and support for grandparent caregivers living with chronic conditions.
